# Telemonitoring in fasting individuals with Type 2 Diabetes Mellitus during Ramadan: A prospective, randomised controlled study

**DOI:** 10.1038/s41598-017-10564-y

**Published:** 2017-08-31

**Authors:** Jun Yang Lee, Chee Piau Wong, Christina San San Tan, Nazrila Hairizan Nasir, Shaun Wen Huey Lee

**Affiliations:** 1 School of Pharmacy, Monash University Malaysia, Jalan Lagoon Selatan, 47500 Bandar Sunway, Selangor Darul Ehsan, Malaysia; 2 Jeffrey Cheah School of Medicine and Health Sciences, Monash University Malaysia, Jalan Lagoon Selatan, 47500 Bandar Sunway, Selangor Darul Ehsan, Malaysia; 3 School of Allied Health Sciences, SEGi University, 9 Jalan Teknologi, Taman Sains Selangor, PJU 5, Kota Damansara, 47810 Petaling Jaya, Selangor Darul Ehsan, Malaysia; 4 Klinik Kesihatan Putrajaya, Jalan P9g1, Presint 9, 62250, Putrajaya, Wilayah, Persekutuan Putrajaya Malaysia; 5 Asian Centre for Evidence Synthesis in Population, Implementation and Clinical Outcomes (PICO), Health and Well-being Cluster, Global Asia in the 21st Century (GA21) Platform, Monash University Malaysia, Bandar Sunway, Selangor Malaysia

## Abstract

We determined the impact of a remote blood glucose telemonitoring program with feedback in type 2 diabetes mellitus patients fasting during Ramadan compared to conventional self-monitoring method. A twelve-week cluster randomised study, with 85 participants who wish to fast for at least 15 days during Ramadan was conducted. Self-measurement and transmission of blood glucose results were performed six times daily during Ramadan. Results were transmitted to a secure website for review with feedback from case manager if necessary. The control group received usual care. The main outcome was the number of participants experiencing hypoglycaemia during Ramadan and at the end of the study. During Ramadan, the number of participants reporting hypoglycaemia was significantly lower in the telemonitoring group [Odds ratio (OR): 0.186, 95% confidence interval: 0.04–0.936; p = 0.04]. Similarly, the proportion of participants reporting symptomatic hypoglycaemia at the end of the study was significantly lower in the telemonitoring group (OR: 0.257, 95% CI: 0.07–0.89; p = 0.03). A reduction of 1.07% in glycated haemoglobin levels was observed in the telemonitoring group compared to 0.24% in the control group (p < 0.01). Overall, telemonitoring was a useful adjunct to reduce the risk of hypoglycaemia during Ramadan with no deterioration in glycaemic control

## Introduction

Islam is the second largest religion in the world, with a total of 1.5 billion followers^1^. As part of the Islamic faith practice, Muslims are required to observe the month of Ramadan, which falls on the 9th lunar month of the Islamic calendar. During this time, it is an obligatory duty for all healthy Muslims to fast (completely abstain from food and water) from sunrise, known as ‘Suhur’ to sunset, known as ‘Iftar’. Exemptions are permitted for individuals who are ill, pregnant, travelling or present an overall weakness that could lead to complications when fasting^2, 3^. Even so, many choose to fulfil their religious duty and continue to fast during this month despite discouragement from doctors and treatment guidelines^4–6^.

In the Epidemiology of Diabetes and Ramadan (EPIDIAR) study, which collected information on Muslim population in 13 countries, the study found that 78% of those with type 2 diabetes (T2DM) choose to fast for at least 15 days during Ramadan^7^. Approximately half of the EPIDIAR study population did not change their lifestyle such as physical activities, sleep duration, and food or fluid intake during Ramadan, which increases the risk of hypoglycaemia. The study reported a 7.5-fold increase in severe hypoglycaemia in T2DM fasting Muslims. It also highlighted several challenges and opportunities to improve the management of diabetes during Ramadan, including the provision of diabetes-focused education as well as changing the patient’s diabetes regimen to minimise the risks of hypoglycaemia during this period^7^.

The use of telemedicine to manage patients with chronic conditions has been shown to be promising as it provides an opportunity for both patients and healthcare providers to be involved in care management and provides the ability to respond promptly to any of the patients needs^8–10^. Several studies have shown that telemedicine could improve HbA1c levels and other secondary parameters related to diabetic complications^11–13^. However, to date, only a few studies have examined the potential of telemedicine in reducing hypoglycaemia, which is the most important issue for T2DM patients; most notably during Ramadan^14^. In this study, we examine the effects of a telemedicine program on patients fasting during Ramadan.

## Methods

The Making Ramadan Fasting A Safer Experience (MRFAST) study was a 12-week cluster-randomised controlled trial comparing telemedicine with the usual general practice care among patients with T2DM who wish to fast during Ramadan.

### Study design

Eligible participants were identified from public primary health care practices in the Klang district from April to June 2015. All 11 public health clinics in the geographical area were invited to participate and five agreed to participate. A researcher, independent of the study team, conducted the cluster randomisation and allocated clinics to telemonitoring intervention (TG) or usual care (UC) group using a centrally administered treatment code. All patient baseline assessments in the practice were completed before allocation was revealed.

In each participating clinic, the nurses generated a list of eligible participants through the extraction of patient’s medical file and primary data collection. Participants were eligible for inclusion if they were aged >18 years with non-insulin dependent type 2 diabetes mellitus diagnosis (minimum 6 months diagnosis), their most recent glycated haemoglobin (HbA1c) levels were between 7.5% and 11.0% (58–96 mmol/mol), they had access to the Internet, an email address, and smartphone with 3 G services and expressed an intention to fast during Ramadan (minimum 15 days fasting). We excluded participants who were unwilling or unable to provide informed consent, if they had a complex debilitating medical condition (e.g. severe mental disorder or life-threatening illness), those who were pregnant or nursing, those who planned to relocate to another study location during the study period or those who lacked the support from primary care provider and caretakers.

All eligible participants were informed of the purpose of the study and invited to participate in the study. Participants who expressed interest were subsequently requested to make an appointment with the participating clinics and written informed consent was obtained from each participant prior to baseline examination. The Medical Research and Ethics Committee, Malaysia (NMRR-14–177–19466) and Monash University Research Ethics Committee (CF14/1977–2014001016) approved this study. This study was registered with Clinicaltrial.gov: NCT02189135 (Registration date: 9^th^ July 2014). The methods performed were conducted in accordance with the approved guidelines.

### Intervention

The intervention comprised of a multi-faceted diabetes management program conducted in collaboration with a multi-disciplinary health care team consisting of pharmacists, physicians, and nurses. A web-enabled glucometer (MyGlucoHealth, Entra Health System, San Diego, California), a mobile diabetes software application and a web portal were devices used in the intervention. The mobile software allowed participants in the TG to upload their glucose reading data via Bluetooth on their mobile phone, which will be recorded, saved and transmitted to an online portal to be viewed by the researcher. The web portal was augmented to the mobile application device and participants email address to provide a reminder to participants who had to measure their glucose level regularly. Additionally, this system would automatically generate a message alert to inform the researcher in the event that there were 3 consecutive hypoglycaemic reading during this study (≤3.9 mmol/L). All participants and their physicians were able to access the medical records, and the case manager would contact participants to provide advice on diabetes management, medication adherence, and lifestyle modification. All participants were requested to measure blood glucose six times daily during the Ramadan period, or more often if they wished.

### Control

Participants allocated to UC were asked to continue to attend their regular clinic check according to usual routine practice that was based on the clinical practice guideline provided by the Ministry of Health Malaysia^15^. They were also referred to the nurse or dietician if deemed necessary.

### All participants

To standardise the education levels, all participants were provided with a Ramadan-focused education that included lifestyle and medication management advice. Education sessions were provided to accommodate participants’ schedule; hence individual as well as combined orientation education sessions were conducted to ensure all participants received adequate education before Ramadan. Participants were given supplies including alcohol swabs and disposable test strips for the entire study duration.

### Outcomes

The primary outcome of interest was the rates of hypoglycaemia (symptomatic only and symptomatic with blood glucose levels of ≤3.9 mmol/L) during Ramadan. Hypoglycaemia that required hospitalisation was defined as severe hypoglycaemia^7^. This outcome was chosen based on the results of our pilot study, which found this to be a more pragmatic outcome compared to our original planned outcome of change in serum fructosamine levels^10^. Secondary outcomes were lipid profile (total cholesterol, high-density lipoprotein, low-density lipoprotein and triglycerides), body mass index, and HbA1c level at the end of the 3-month study period. Questionnaires were administered at baseline and at the end of study to assess quality of life, diabetes knowledge, diabetes self-efficacy, and diabetes stress level. We used the Euroqol-5D-3L questionnaire to assess participants quality of life^16^. A 14-item Malaysia version of the Michigan Diabetes Knowledge Test was used to assess participants’ knowledge^17–19^. To assess diabetes distress, we used the 20-item Problem in Diabetes Survey (PAID)^20^ and the 8-item Diabetes Self-Efficacy Scale^21, 22^.

### Sample size

The sample size was calculated based on results of our previous pilot study, which found that the use of telemedicine could reduce the risk of hypoglycaemia in T2DM patients by almost three-quarters^10^. Assuming that the sample size with an 80% power is required to detect a 25% difference in rates of hypoglycaemia between both groups (one-sided α = 0.05), we would need an estimated of 66 respondents. Taking into consideration an allowance of a 20% dropout due to loss during follow-up and discontinuation of the program, we would need a minimum of 80 patients in the study.

### Statistical analysis

Descriptive analysis was performed for all variables. For unadjusted comparison between study groups, student’s t-test was used for continuous variables while chi-square test was used for discrete variables. All data were analysed based upon an intention-to-treat basis using a complete case analysis with the assumption that missing outcomes are missing at random. Linear mixed effects models with random intercept were used to compare the mean changes in the primary and secondary clinical measurement outcomes as well as for the binary outcomes of hypoglycaemia at weeks 4 and 12 between the groups. Within-practice and within patient correlation were measured using the random effects model. A p-value of less than 0.05 was considered significant. All statistical analysis was performed using IBM SPSS Statistics version 20 (Armonk, NY).

### Data availability statement

Data generated and analysed in this study is available from the corresponding author on reasonable request.

## Results

**Figure 1:**
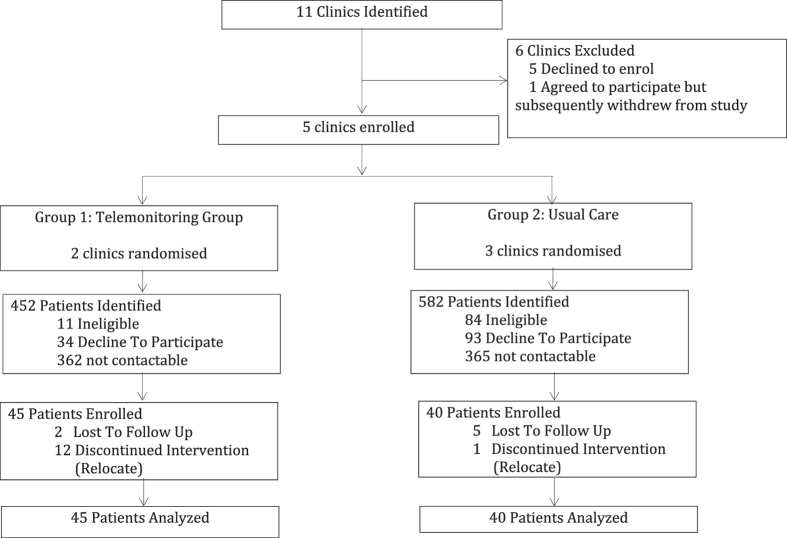
Study Flow Diagram.

Of the 1,034 identified participants who were assessed for eligibility, 85 participants consented to participate with 45 assigned to the telemonitoring group and 40 assigned to the usual care group (Fig. 1). At baseline, participants had a mean age of 53.5 years, with a body mass index of 29.7 kg/m^2^ and HbA1c level of 8.7% (72 mmol/mol). Most of the baseline characteristics were relatively balanced between both groups, but participants in the usual care group had a longer history of diabetes disease (p = 0.05). Participants in the telemonitoring intervention group reported higher levels of fasting plasma glucose at baseline (p = 0.02; Table 1).

**Table 1:** Baseline demographics and clinical characteristics of participants.

Characteristic	Usual Care	Telemonitoring	*p-v*alue^a^
	(n = 40)	(n = 45)	
Men, *n*(%)^b^	16 (40.00)	24 (60.00)	0.21
Women, *n*(%)^b^	24 (60.00)	21 (40.00)	
Age (years), mean (±SD)	53.77 (8.03)	53.24 (7.29)	0.89
Duration of diabetes since diagnosis(*years*), mean (±SD)	10.04 (7.64)	7.91 (4.81)	0.05
Education,*n*(%)^b^			
None	3 (7.50)	1 (2.20)	0.80
Primary	13 (32.50)	12 (26.70)	
Secondary	22 (55.00)	30 (66.70)	
Tertiary	2 (5.00)	2 (4.40)	
Employment status,*n*(%)^b^			
Employed	19 (47.50)	25 (55.60)	0.78
Unemployed	20 (50.00)	20 (44.40)	
Marital status, *n* (%)^b^			
Married	36 (90.00)	42 (93.30)	0.83
Divorced	3 (7.50)	2 (4.40)	
Widower	1 (2.50)	1 (2.20)	
Weight (kg), mean (±SD)	77.14 (13.19)	71.67 (13.30)	0.12
BMI (*kg/m* ^2^), mean (±SD)	30.28 (5.05)	29.20 (5.98)	0.56
Systolic blood pressure (mmHg),			
mean (±SD)	133.50 (33.12)	132.48 (20.73)	0.87
Diastolic blood pressure (mmHg), mean (±SD)	84.17 (16.49)	85.57 (15.66)	0.97
Serum fructosamine (µmol/L), mean (±SD)	337.34 (62.41)	335.32 (78.73)	0.46
HbA1c (%), mean (±SD)	8.79 (1.15)	8.69 (1.12)	0.16
FPG (mmol/L), mean (±SD)	8.68 (3.30)	9.46 (3.01)	0.02
Total Cholesterol (mg/dL), mean (±SD)	4.87 (0.89)	4.74 (0.72)	0.10
Triglycerides (mg/dL), mean (±SD)	2.07 (1.54)	2.05 (0.79)	0.38
HDL (mg/dL), mean (±SD)	1.16 (0.32)	1.31 (0.42)	0.41
LDL (mg/dL), mean (±SD)	2.81 (0.94)	2.49 (0.75)	0.40
EQ-5D, mean (±SD)	0.75 (0.35)	0.80 (0.24)	0.25
Diabetes Knowledge Test, mean (±SD)	32.85 (18.48)	38.41 (12.30)	0.06
Diabetes Distress (PAID), mean (±SD)	1.57 (0.78)	1.60 (0.80)	0.77
Diabetes Self-Efficacy Scale), mean (±SD)	3.97 (2.59)	4.2 (2.58)	0.73

^a^ *P* values based on independent *t* test. ^b^Chi-squared tests were used SD, standard deviation; BMI, body mass index; FPG, fasting plasma glucose; HDL, high density lipoprotein; LDL, low density lipoprotein; EQ. 5D, EuroQoL-5D; PAID, Problem in Diabetes Survey

At the end of the 3-month study period, 13 participants had discontinued treatment as they had moved out of the study geographical area while another 7 participants were lost in the follow-up process (Fig. 1). Most clinical parameters except lipid profiles were comparable between participants who discontinued treatment and participants who have completed the study (Table 2).

**Table 2:** Demographic comparison between completers and non-completers of the study.

Characteristic	Completers	Non Completers	*p-v*alue^a^
	(n = 65)	(n = 20)	
Men, *n* (%)^b^	35 (53.80)	5 (25.00)	0.02
Women, *n* (%)^b^	30 (46.20)	15 (75.00)	
Age (years), mean (±SD)	53.74 (7.09)	52.70 (9.25)	0.58
Duration of diabetes since diagnosis (*years*), mean (±SD)	8.71 (6.65)	9.55 (5.38)	0.61
Education, *n* (%)^b^			
None	2 (3.00)	1 (5.00)	0.98
Primary	19 (29.30)	6 (30.00)	
Secondary	41 (63.00)	12 (60.00)	
Tertiary	3 (4.60)	2 (5.00)	
Employment status, *n* (%)^b^			
Employed	30 (46.20)	9 (45.00)	0.57
Unemployed	35 (53.80)	11 (55.00)	
Marital status, *n* (%)^b^			
Married	60 (92.30)	18 (90.00)	0.50
Divorced	3 (4.60)	2 (10.00)	
Widower	2 (3.00)	0 (0.00)	
Weight (kg), mean (±SD)	75.08 (13.43)	71.51 (13.51)	0.40
BMI (*kg/m* ^2^), mean (±SD)	29.89 (5.77)	29.13 (4.88)	0.71
Systolic blood pressure (mmHg),mean (±SD)	136.42 (23.88)	132.15 (27.01)	0.50
Diastolic blood pressure (mmHg), mean (±SD)	85.86 (13.26)	87.40 (13.76)	0.65
Serum fructosamine (µmol/L), mean (±SD)	341.77 (58.87)	354.01 (53.03)	0.41
HbA1c (%), mean (±SD)	8.25 (1.61)	8.42 (1.10)	0.65
FPG (mmol/L), mean (±SD)	8.66 (2.62)	9.73 (2.77)	0.12
Total Cholesterol (mg/dL), mean (±SD)	4.81 (0.82)	4.51 (0.47)	0.13
Triglycerides (mg/dL), mean (±SD)	1.89 (0.69)	2.70 (1.86)	<0.01
HDL (mg/dL), mean (±SD)	1.19 (0.33)	1.02 (0.17)	0.03
LDL (mg/dL), mean (±SD)	2.68 (0.81)	2.20 (0.86)	0.02
EQ-5D, mean (±SD)	0.82 (0.15)	0.86 (0.10)	0.08
Diabetes Knowledge Test, mean (±SD)	40.00 (15.95)	41.07 (13.88)	0.83
Diabetes Distress (PAID), mean (±SD)	1.58 (0.78)	1.60 (0.82)	0.83
Diabetes Self-Efficacy Scale, mean (±SD)	4.01 (2.67)	4.35 (2.27)	1.00

^a^ *P* values based on independent *t* test. ^b^Chi-squared tests were used SD, standard deviation; BMI, body mass index; FPG, fasting plasma glucose; HDL, high density lipoprotein; LDL, low density lipoprotein; EQ. 5D, EuroQoL-5D; PAID, Problem in Diabetes Survey.

### Risk of hypoglycaemia

**Figure 2:**
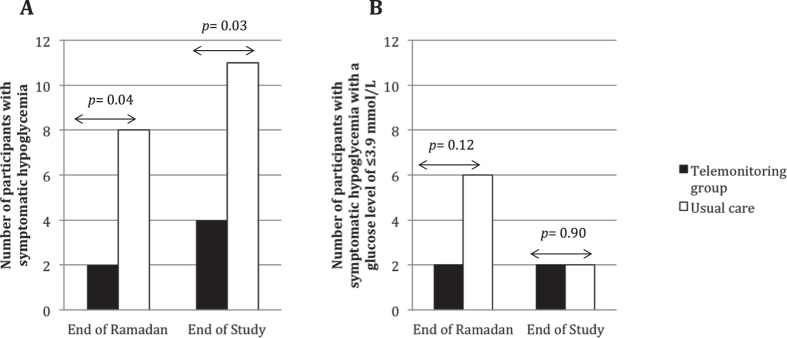
The number of participants reporting hypoglycaemia at the end of Ramadan and end of the study. Proportion of participants reported symptomatic hypoglycaemia (**A**) and proportion of participants reported symptomatic hypoglycaemia with a glucose level of ≤3.9 mmol/L (**B**) comparing telemonitoring versus usual care are shown.

Figure 2 shows the proportion of participants who had reported hypoglycaemia at the end of Ramadan and at the end of the study. At the end of the Ramadan period, reported symptomatic hypoglycaemia was lower in participants in the telemonitoring group (n = 2/45) compared to the usual care group (n = 8/40) [Odds ratio (OR): 0.186, 95% confidence interval: 0.04–0.936; p = 0.04)]. However, only 2 participants in the telemonitoring group and 6 participants in the usual care group reported symptomatic hypoglycaemia with a glucose level of ≤3.9 mmol/L (OR: 0.26, 95% CI: 0.05–1.39; p = 0.12).

Fewer participants in the telemonitoring group (n = 4) had also reported symptomatic hypoglycaemia compared to usual care group (n = 11) at the end of the 3-month intervention (OR: 0.257, 95% CI: 0.07–0.89; p = 0.03). Two participants each in the telemonitoring group and the usual care group reported symptomatic hypoglycaemia with a glucose level of ≤3.9 mmol/L (OR: 0.88, 95% CI: 0.11–6.58; p = 0.90). No severe hypoglycaemic episode was reported throughout the entire study period.

### Secondary outcomes

Table 3 showsthe mean (SD) changes in biomedical outcomes of the study groups at the end of 3 months. There was a significant decrease in mean HbA1c from 8.69% to 7.62% in the telemonitoring group compared to a decrease from 8.79% to 8.55% in the usual care group at the end of the study (p < 0.01). More participants in the telemonitoring group achieved HbA1c level of ≤ 7.0% (15 participants) compared to the usual care group (12 participants) (OR: 1.11, 95% CI: 0.46–2.65; p = 0.81). The telemonitoring group also reported significant improvements in lipid control at the end of Ramadan. An improvement in the quality of life was also noted, albeit not achieving statistical significance (p = 0.06). No significant changes in other secondary outcome measures including blood pressure, weight, diabetes distress assessment, and diabetes self-efficacy scale were noted.

**Table 3:** Primary and secondary study outcomes.

Outcome	Telemonitoring group (TG) (n = 45)	Reduction from baseline	Usual care group (UC) (n = 40)	Reduction from baseline	p-value		Difference change from baseline	p-value
	Mean (SD)	Mean (SD)	Mean (SD)	Mean (SD)	TG	UC	Mean (SD)	
Serum fructosamine (µmol/L)								
End of Ramadan	333.28 (74.90)	−0.78 (57.41)	336.92 (82.73)	2.10 (30.89)	0.93	0.65	−2.88 (70.68)	0.78
Serum HbA1c (%)								
End of Study	7.62 (1.61)	−1.06 (1.66)	8.55 (1.86)	−0.22 (1.48)	<0.01	0.33	−0.84 (1.82)	<0.01
Fasting plasma glucose (mmol/L)								
End of Ramadan	9.72 (3.00)	0.27 (2.64)	8.96 (3.87)	0.25 (2.80)	0.62	0.89	0.02 (3.98)	0.97
End of Study	9.69 (3.56)	−0.01 (3.57)	8.75 (3.83)	0.06 (3.42)	0.93	0.69	−0.07 (3.96)	0.97
Total cholesterol (mg/dL)								
End of Ramadan	4.46 (1.07)	−0.31 (0.81)	4.90 (0.97)	0.03 (0.48)	<0.01	0.35	−0.34 (0.94)	0.02
End of Study	4.19 (1.30)	−0.60 (1.17)	5.00 (0.98)	0.12 (1.07)	0.04	0.69	−0.72 (1.73)	0.01
Triglycerides (mg/dL)								
End of Ramadan	1.78 (0.82)	−0.22 (0.44)	2.11 (1.66)	0.04 (0.54)	0.08	0.95	−0.26 (0.84)	0.05
End of Study	1.84 (0.87)	−0.15 (0.66)	2.07 (1.57)	−0.01 (0.72)	0.41	0.69	−0.14 (0.91)	0.29
High density lipoprotein (mg/dL)								
End of Ramadan	1.53 (0.56)	0.22 (0.47)	1.17 (0.34)	0.01 (0.09)	0.11	<0.01	0.21 (0.48)	<0.01
End of Study	1.44 (0.51)	0.12 (0.59)	1.27 (0.41)	0.10 (0.28)	0.17	0.01	0.02 (0.63)	0.84
Low density lipoprotein (mg/dL)								
End of Ramadan	2.94 (0.96)	0.45 (0.70)	2.89 (0.98)	0.08 (0.62)	0.03	0.39	0.37 (0.69)	<0.01
End of Study	2.78 (1.01)	0.25 (0.91)	2.90 (0.83)	0.09 (0.98)	0.08	0.49	0.16 (1.30)	0.42
Systolic blood pressure (mmHg)								
End of Ramadan	133.57 (10.08)	1.09 (23.07)	129.05 (22.16)	−3.95 (24.96)	0.11	0.74	5.04 (34.98)	0.33
End of Study	136.88 (9.05)	4.40 (22.63)	132.40 (22.47)	−0.97 (24.73)	0.23	0.32	5.37 (34.10)	0.29
Diastolic blood pressure (mmHg)								
End of Ramadan	78.77 (1.27)	−6.80 (15.75)	76.72 (22.16)	−6.62 (9.13)	<0.01	<0.01	−0.18 (17.76)	0.94
End of Study	79.40 (1.49)	−6.20 (15.78)	77.22 (22.47)	−6.17 (9.07)	0.74	0.67	−0.02 (17.93)	0.99
Weight (kg)								
End of Ramadan	71.38 (13.27)	−0.28 (1.06)	77.02 (13.44)	−0.10 (0.87)	0.07	0.42	−0.18 (1.53)	0.43
End of Study	72.21 (13.13)	0.82 (0.54)	77.66 (13.40)	0.56 (0.76)	0.01	0.01	0.26 (0.91)	0.06
BMI (*kg/m* ^2^)								
End of Ramadan	29.08 (5.96)	−0.11 (0.42)	30.23 (5.14)	−0.04 (0.34)	0.07	0.40	−0.07 (0.60)	0.42
End of Study	29.42 (5.92)	0.33 (0.22)	30.49 (5.11)	0.22 (0.30)	0.01	0.02	0.11 (0.37)	0.05
EQ-5D								
End of Study	0.87 (0.11)	0.002 (0.04)	0.81 (0.26)	−0.03 (0.10)	0.78	0.61	0.03 (0.10)	0.06
Diabetes knowledge test^17–19^*								
End of Study	68.41 (14.95)	29.82 (20.19)	64.11 (16.05)	27.14 (19.15)	0.12	0.84	2.68 (25.30)	0.50
Diabetes Distress Scale (PAID)								
End of Study	1.66 (0.69)	−0.40 (0.84)	1.51 (0.67)	−0.40 (0.95)	0.53	0.57	0.00 (1.33)	1.00
Diabetes Self-Efficacy Scale								
End of Study	4.55 (2.42)	0.40 (3.82)	4.2 (2.35)	0.22 (3.86)	0.54	0.71	0.18 (6.01)	0.855

*Score range (0–100). Higher score indicates better diabetes knowledge.

BMI; Body Mass Index; PAID, Problem in Diabetes Survey.

## Discussion

To our knowledge, this was the largest cluster-randomised study that used telemonitoring intervention in diabetes management during Ramadan in a community setting. Our previous pilot study found participants in the telemonitoring group reported fewer hypoglycaemic events compared to usual care group although clinical significant differences were not found^10^. Similarly, in this study, we found that telemonitoring coupled with diabetes education resulted in fewer participants in the telemonitoring group experiencing hypoglycaemia at the end of Ramadan and at the end of the study as well as statistically significant improvements in glycaemic control amongst Muslim patients with poor glycaemic control. Mean HbA1c levels in the telemonitoring group improved by 1.07% compared with 0.24% for usual care group at the end of the study. Diabetes education was also found to be able to improve the patients’ quality of life at the end of the study.

In addition, we also noted improvements in lipid levels in the telemonitoring group. This could be due to a combination of our intervention as well as fasting since participants typically consumed only two meals a day during Ramadan, which might have resulted in a lower caloric intake that could contribute to the lower lipid levels. Likewise, a sudden increase in caloric intake after the fasting month could explain the increase in weight at the end of the study. Additionally, improvement in diabetes education could be a contributing factor to the improvements in lipid levels. We also observed an increase in fasting plasma glucose levels during the study at the end of Ramadan. This could possibly be due to the increased intake of refined sugars contained in local delicacies which were popular during the Ramadan period in this region^23^. Although diabetes knowledge of participants had improved, nevertheless the act of breaking fast during Ramadan was typically considered a family affair^24^, hence; dietary indiscretion could be difficult during the fasting month. Although a correlation was found between serum fructosamine and fasting plasma glucose, however, its sensitivity to predict glycaemic changes was low^25^, which was reflected in our findings. Low albumin concentration and increased albuminuria were possible contributors to the low sensitivity^26^.

Few studies in diabetes management explored the use of telemonitoring as a means of intervention during Ramadan even though many have reported its effectiveness in diabetes management^12, 13, 27, 28^. Our findings were in line with the recent recommendation for diabetes management that had encouraged patient engagement and lifestyle change for better improvement in blood glucose control^29^. Although improvements in diabetes distress, self-efficacy, diabetes knowledge, blood pressure, and weight change were not convincing, our finding suggested that patient education coupled with telemonitoring could be beneficial to individuals who fast during Ramadan.

Additionally, there was evidence to show that structured patient education, especially during Ramadan, could reduce the risk of hypoglycaemia while maintaining glycaemic control in patients^14^. In our current study, we noted that the rates of hypoglycaemia are much lower with no reports of severe adverse hypoglycaemic incidences. This phenomenon could be attributed to baseline diabetes education, which was provided to all participants taking part in the study. Some healthy aspects, which were included in the education, were principles of nutrition, effects of different time and dosing of oral hypoglycaemic agents as well as exercises suitable to be performed during Ramadan, might have empowered our participants with the skills to prevent and recognise early hypoglycaemic symptoms.

In summary, our study achieved our goal to reduce the risk of hypoglycaemia and control of glycaemic levels during Ramadan. Individuals who wish to fast should undergo a pre-Ramadan assessment with their physicians, with particular attention paid to older aged patients. Assessment should include functional capacity as well as cognition levels in order to evaluate the risk of fasting and there is a need to individualise any therapy to adapt to the patients’ needs. It might also be prudent to advise these individuals to test fasting a few weeks before Ramadan in order to assess any potential risk of hypoglycaemia, and to encourage regular self-monitoring blood glucose (SMBG) especially if they were on medications such as sulfonylureas. If needed, the use of incretin-based therapies could be considered due to the lower risk of hypoglycemia^14^. Indeed, the recent International Diabetes Federation guidelines recommended that physicians should provide pre-Ramadan advice one or two months prior to Ramadan fasting^30^. It also recommended a risk assessment, which included evaluating patients’ regular glycaemic control, medication adherence, age and geographical region. To ensure a safer fasting experience more emphasis need to be placed on the importance of patients’ health assessment before fasting and therefore, more collaborations should be carried out between physicians and religious teachers, “Imams”.

Our study had several strengths. We had a large sample size. Participants were recruited from public health clinics, which catered to nearly three-fifth of the population in Malaysia^31^, and thus was more representative than studies that were conducted in specialist and research centres.

This study did have some limitations, which warranted some discussion. Due to the nature of the study, blinding of participants was not possible; hence a cluster-randomisation method was used to minimise cross-contamination between study groups. Most participants in the telemonitoring group were older and had a lower level of education. This necessitated the researchers to guide participants multiple times during the course of the study on the use of the devices. As with most complex interventions, we could not separate the intervention effects attributable to the case management or telemonitoring. Additionally the utilisation of this technology was provided at no cost to the participants. Further studies are required to explore the cost of utilising such technology in the current healthcare setting. Furthermore, our study may not be generalizable as the intervention in this study was conducted in a specific religious group thus did not represent the whole multi-ethnic population of Malaysia. We hope to address these issues when our next planned study, which has a larger sample size, with a longer-term follow-up, is completed^32^.

In conclusion, we observed that it was practical to implement Ramadan-specific education coupled with telemonitoring as a supplement to current diabetes care management for individuals who fast during this period. Individuals with type 2 diabetes mellitus who intend to fast should undergo a medical assessment at least 1 month prior to the month of Ramadan and receive an education that is tailored towards Ramadan fasting. A remote alert that reports low hypoglycaemic levels should be implemented to allow appropriate intervention should a hypoglycaemic event occurs.
